# Infantile orbital abscess caused by methicillin-resistant *Staphylococcus aureus*: a case report and literature review

**DOI:** 10.3389/fped.2023.1272852

**Published:** 2023-12-22

**Authors:** Yanran Qin, Junming Huo, Chengjun Liu, Yueqiang Fu, Jing Li

**Affiliations:** Department of Intensive Care Unit, Children's Hospital of Chongqing Medical University, National Clinical Research Center for Child Health and Disorders, Ministry of Education Key Laboratory of Child Development and Disorders, China International Science and Technology Cooperation Base of Child Development and Critical Disorders, Chongqing Key Laboratory of Pediatrics, Chongqing, China

**Keywords:** infant, orbital abscess, MRSA, vancomycin, corticosteroids

## Abstract

**Objective:**

To report and review infantile orbital abscess caused by methicillin-resistant *Staphylococcus aureus* (MRSA).

**Methods:**

We report a case of MRSA-induced infantile orbital abscess accompanied by sepsis, pneumonia, and purulent meningitis. We systematically review cases of MRSA-induced infantile orbital abscess published in PubMed, Web of Science and ScienceDirect until April 2023.

**Results:**

We reviewed 14 patients [our patient + 13 patients (10 papers) identified via literature searches]. There were nine boys and five girls; nine neonates and five older infants; and 8 full-term births and 1 preterm birth. The gestational age at birth was unknown for five infants. The right and left orbits were affected in 10 and 4 patients, respectively. The clinical presentation included periorbital soft-tissue edema or redness (11 patients), fever (7 patients), exophthalmos (10 patients), limited eye movement (4 patients), purulent eye secretions (2 patients), and skin abscess and convulsion (1 patient each). The source of infection was sinusitis (8 patients), vertical transmission, gingivitis, dacryocystitis, upper respiratory tract infection (1 patient each), and unknown (2 patients). MRSA was detected in blood (6 patients) or pus culture (8 patients). Vancomycin or linezolid were used for 11 patients; corticosteroids were administered to only 1 patient. Surgical drainage was performed for 13 infants (external drainage, 11 patients; endoscopic drainage, 2 patients). Two patients initially had pulmonary and intracranial infections. Except for one patient with neurological dysfunction at discharge, all other infants had no sequelae or complications.

**Conclusion:**

Early aggressive anti-infective treatment and timely drainage are essential for managing MRSA-induced infantile orbital abscess.

## 1. Introduction

Infantile orbital abscess is a rare, severe, and intractable ocular infection, which tends to progress rapidly. The most common causes of orbital abscess include upper respiratory tract infection, dacryocystitis, sinusitis, gingivitis, and vertical maternal transmission (1). Orbital abscess is characterized by fever, eyelid swelling, exophthalmos, and restricted eye movement. It can be definitively diagnosed using ocular imaging examinations such as computed tomography (CT), magnetic resonance imaging (MRI), and ultrasonography. Severe orbital abscess may result in visual impairment, intracranial infection, and even visual sequelae and nervous system dysfunction. Hence, timely diagnosis and treatment are critical to improve prognosis.Ocular infections are less commonly observed in infants and children compared to adults. Currently, the most common bacteria responsible for causing infantile orbital abscess are *Streptococcus pneumoniae*, *Streptococcus pyogenes*, *Haemophilus influenzae*, and *Staphylococcus aureus*. With the introduction of vaccines for *Streptococcus pneumoniae* and *Haemophilus influenzae*, cases of infection with *Staphylococcus aureus*, notably methicillin-resistant *Staphylococcus aureus* (MRSA), have dramatically increased (2). MRSA, which was first described in 1961 in the United Kingdom, poses a significant clinical challenge due to its resistance to beta-lactam antibiotics. MRSA infection can lead to complications and sequelae. Although MRSA has become more prevalent among infants with orbital abscess, it remains a rare cause of infantile orbital abscess, as compared to other bacteria.This case report describes an infant with MRSA-induced orbital abscess caused by sepsis, with concomitant pneumonia and meningitis. We also conducted a literature review to analyze and summarize the treatment of infantile orbital abscess caused by MRSA.

## 2. Materials and methods

### 2.1. Case report

**Figure 1:**
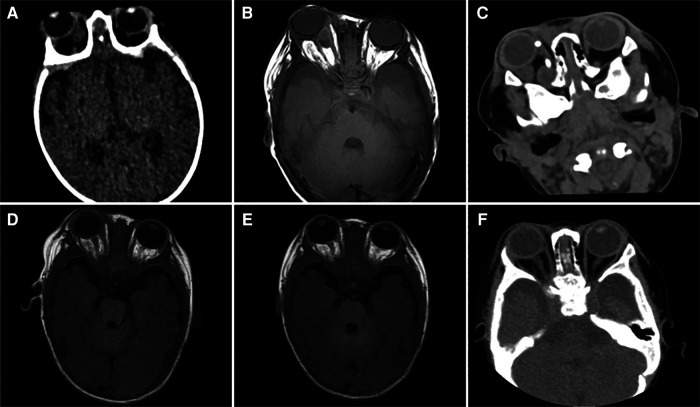
(**A**) An ocular computed tomography (CT) scan performed on the 1st day of hospitalization reveals a shadow with soft-tissue density in the right orbit. (**B**) Ocular magnetic resonance imaging (MRI) conducted on the 4th day of hospitalization displays irregular cystic lesions located behind the right eyeball and within the optic nerve canal. (**C**) An ocular CT scan performed on the 9th day reveals a reduction in the size of the lesion within the right orbit compared to the preoperative condition. (**D**) An ocular MRI conducted on the 19th day of hospitalization shows a notable reduction in the size of the initial irregular cystic lesion. (**E**) An ocular MRI conducted on the 31st day reveals the near-complete disappearance of the original lesion. (**F**) Ocular CT performed on the 65th day shows that no abscess remains in the right orbit.

**Figure 2:**
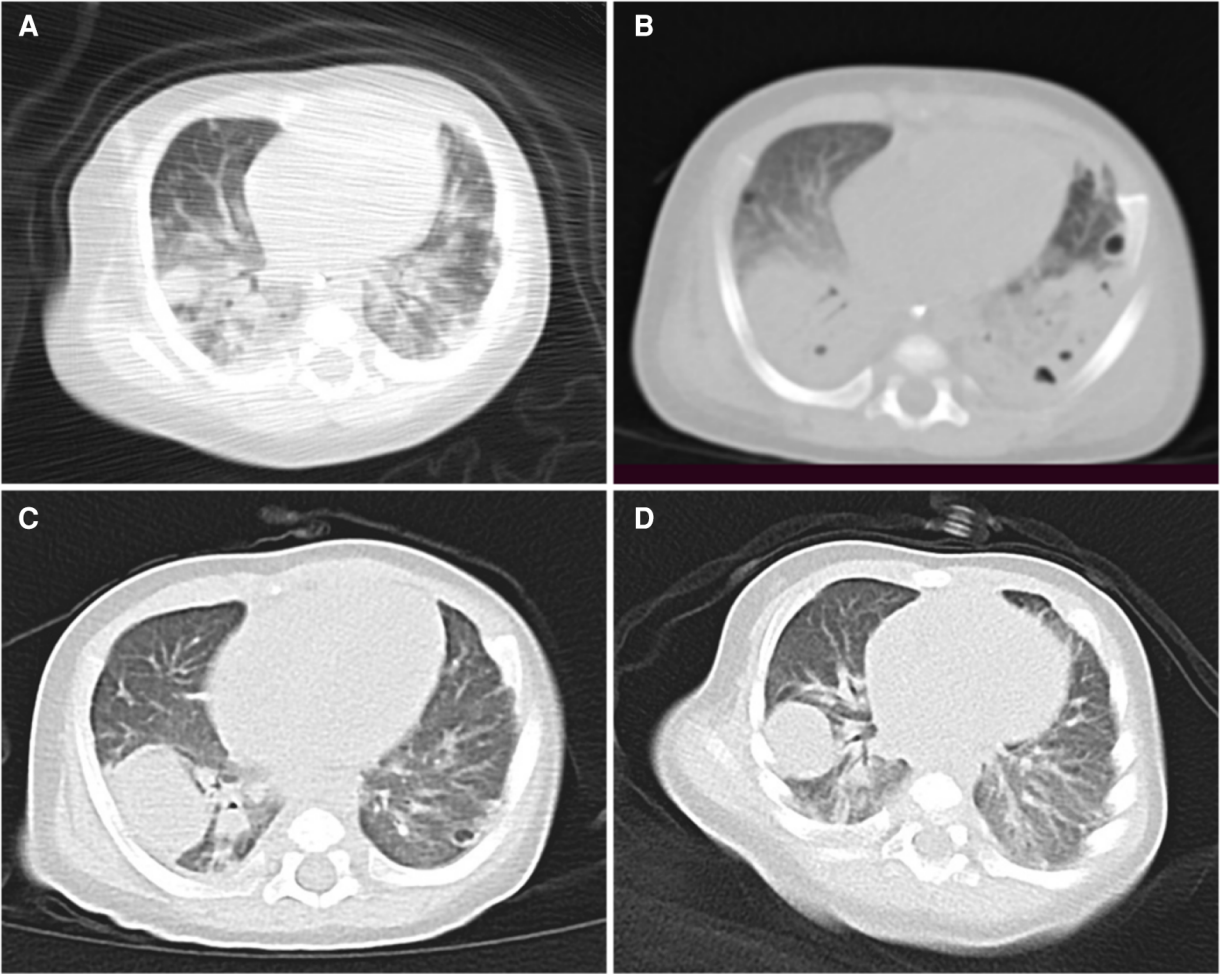
(**A**) Lung computed tomography (CT) performed on the 1st day of hospitalization shows scattered dense shadows in both lungs, accompanied by a few cystic translucent shadows. (**B**) Lung CT conducted on the 9th day shows multiple nodules and capsular shadows in both lungs that are more obvious than before. (**C**) Lung CT performed on the 22nd day shows that the lesions in both lungs are less prominent than before. (**D**) Lung CT performed on the 35th day shows further improvement in both lungs.

**Figure 3:**
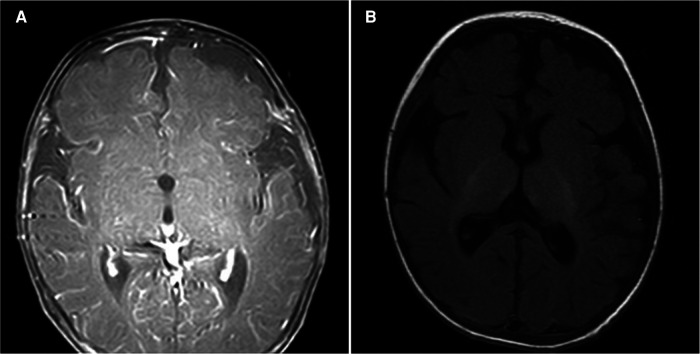
(**A**) Magnetic resonance imaging (MRI) of the head performed on the 4th day of hospitalization shows obvious meningeal enhancement. (**B**) Head MRI on the 19th day shows that the meningeal enhancement is less obvious than before.

A 66-day-old full-term male infant, weighing 7 kg, presented at Chongqing Children's Hospital with a history of intermittent fever since 7 days and swelling of the right eyeball since 5 days. Upon admission, a physical examination revealed a febrile condition with a body temperature of 38.9°C. His respiratory rate was 70 breaths/min, with a heart rate of 180 beats/min, blood pressure of 70/46 mmHg, and oxyhemoglobin saturation of 93% (without oxygen inhalation). Although the infant was conscious, he displayed signs of irritability. No skin rash or purulent lesions were observed. The infant's right eye was bulging, and showed limited movement and a small amount of secretion in the medial corner. Additionally, no eyelid retraction was noted, and coarse moist rales were apparent in both lungs. Laboratory examinations revealed an elevated white blood cell count of 44.52 × 10^9^/L, a high C-reactive protein (CRP) level of 182.97 mg/L, and a procalcitonin level of 7.98 ng/ml. Cerebrospinal fluid (CSF) analysis showed a total cell count of 246 × 10^6^/L, with a white blood cell count of 240 × 10^6^/L. Mononuclear cells constituted 19% and multinuclear cells constituted 81% of the white blood cells detected in the CSF. The concentration of trace proteins in the CSF was 1.53 g/L, that of chloride ions was 115.8 mmol/L, and that of glucose was 1.19 mmol/L. The infant's sputum culture yielded negative results. However, both the blood culture and CSF metagenomic next generation sequencing indicated MRSA. CT examination revealed infection of both lungs and the presence of an orbital abscess. No abnormalities were observed in the ethmoid sinuses (Figures 1A, 2A). MRI of the head confirmed the presence of an orbital abscess and showed slight enhancement of the meninges (Figures 1B, 3A). Abdominal CT and cardiac ultrasound showed no significant infection. The infant had no perinatal abnormalities and no family history of diseases such as immune deficiency. Considering these findings, we diagnosed the infant with MRSA-induced sepsis, orbital abscess, pneumonia, and purulent meningitis.

On the first day of hospitalization, the patient was intravenously administered vancomycin (80 mg q6h) and meropenem (140 mg q8h) for anti-infection treatment. The trough concentration of vancomycin was 16.4 μg/ml. By the 4th day, the patient required the use of a ventilator due to respiratory failure. On the 5th day, the infant's body temperature returned to normal, and he did not show any signs of fever. On the 6th day, external drainage was performed to relieve the right orbital abscess, which was accessed through the inner area of the lower fornix behind the right eyeball. A culture of the collected pus confirmed the presence of MRSA. On the 9th day, a follow-up CT examination indicated a reduction in the size of the orbital lesion compared to the lesion size prior to the drainage (Figure 1C). However, the lesions in both lungs were more prominent, and characterized by the formation of multiple nodules and cavities (Figure 2B). On the 13th day, a second blood culture was sterile. By the 15th day, the infant had been successfully weaned off the ventilator. To address the persistent severe pulmonary infection, vancomycin was changed to linezolid (60 mg q8h). The patient underwent multiple imaging and biochemical examinations, which showed gradual improvement in the intracranial, orbital, and pulmonary infections. However, multiple nodules and cavities were still seen in both lungs (Figures 1D,E, 2C,D, 3B). At 26 days after hospitalization, meropenem was replaced with cefazoxime (300 mg q8h). After another 20 days, cefazoxime was discontinued, and treatment was continued with linezolid alone. On the 53th day, the child was discharged in a healthy condition, with no symptoms or signs, except for a slight cough. Following discharge, the infant continued taking linezolid tablets 60 mg q8h for 2 months. Outpatient follow-up fundus examination and ocular CT at 2 months after discharge showed no abnormalities (Figure 1F).

### 2.2. Literature review

We searched the PubMed, Web of Science and ScienceDirect databases for the following search terms: “MRSA,” “methicillin-resistant Staphylococcus aureus,” “orbital abscess,” “cellulitis,” “subperiosteal abscess,” “cavernous sinus thrombosis,” “children,” “infant,” and “newborn.” Both databases were searched for articles published from the inception of the database to April 2023. The exclusion criteria were as follows: (1) age > 1 year, (2) non-MRSA infection, and (3) no abscess formation in the orbital tissue. We identified a total of 10 papers concerning MRSA-related orbital abscess in 13 infants. We collected the following information about the patients: demographic data (age, gender, and gestational age), position of orbital abscess, clinical manifestations, source of infection, use of antibiotics and corticosteroids, operative approaches, and outcomes. Retrospective statistical analysis was conducted on all gathered data.

## 3. Results

A total of 14 patients (13 patients identified by the literature search plus our patient) with infantile orbital abscess caused by MRSA were included in this literature review. Of the 14 infants, 9 were boys, and 5 were girls. There were 9 neonates and 5 older infants. Eight infants had had full-term births, while one infant was born preterm; the gestational age at birth was unknown for the remaining five infants. The right orbit was affected in 10 infants, while the left orbit was affected in 4 infants. Most infants presented with varying degrees of periorbital soft-tissue edema or redness. Additionally, 10 patients had exophthalmos, 7 patients had fever, 4 patients had limited eye movement, 2 patients had purulent eye secretions, 1 patient had a skin abscess, and 1 patient had convulsion. The source of infection was sinusitis in 8 patients, vertical transmission from the mother in 1 patient, gingivitis in 1 patient, dacryocystitis in 1 patient, upper respiratory tract infection in 1 patient, and unknown in 2 patients. Among the 14 patients, 6 had positive blood culture results for MRSA, while 8 patients only had positive pus culture results. Treatment involved the use of vancomycin or linezolid in 11 patients, and other antibiotics such as rifampicin, clindamycin, and daptomycin in the remaining patients. Corticosteroids were administered to only 1 patient. Surgical drainage was performed for 13 infants, either in the form of external drainage (11 patients) or endoscopic drainage (2 patients). One patient was treated solely with medical treatment and did not require surgery. Upon admission, 2 patients had pulmonary and intracranial infections in addition to the ocular infection, while the remaining infants had isolated ocular infection. At the time of discharge, only 1 patient had neurological dysfunction; the other patients did not experience any sequelae or complications (Table 1).

**Table 1:** Summary of cases of MRSA-induced infantile orbital abscess reported between 2005 and 2021.

Case no.	Author	Year	Age	GAGA	Sex	Side	Clinical manifestation	Infection source	Sample	Antibiotics (duration)	Cortico-steroids	Surgery	Co-infection	Sequela
1	Anari et al. (3)	2005	28 days	34 weeks	M	Right	Fever, surrounding erythema, exophthalmos	Sinusitis	Blood and pus	Flucloxacillin and cefotaxime (unknown), vancomycin and rifampicin (2 weeks)	No	External drainage	No	No
2	Rogers et al. (4)	2007	13 days	Mature	M	Left	Exophthalmos	Sinusitis	Blood	Clindamycin (21 days)	No	Endoscopic drainage	No	No
3	Chung et al. (5)	2011	5 months	Mature	F	Left	Eyelid swelling, chemosis	Sinusitis	Pus	Vancomycin (10 days)	No	External drainage	No	No
4	Vaska et al. (6)	2011	14 days	Mature	F	Right	Fever, eyelid swelling, purulent secretion	Sinusitis	Pus	Flucloxacillin and ceftriaxone (unknown), lincomycin (21 days)	Yes^a^	External drainage	No	No
5	Pérez et al. (7)	2012	13 days	NR	M	Left	Surrounding erythema	Sinusitis	Blood	Vancomycin and rifampicin (3 weeks), sulfamethoxazole (5 weeks)	No	No	No	No
6	Pérez et al. (7)	2012	10 days	NR	M	Left	Chemosis, exophthalmos, convulsion, skin abscess	Sinusitis	Pus	Vancomycin and rifampicin (6 weeks), sulfamethoxazole (unknown)	No	External drainage	Brain abscess, pneumonia	Spastic quadri-plegia
7	Tsironi et al. (8)	2012	28 days	Mature	M	Right	Fever, eyelid swelling, exophthalmos, limited eye movement	Sinusitis	Pus	Daptomycin, rifampicin, ceftriaxone (42 days)	No	External drainage	No	No
8	Lin et al. (9)	2013	28 days	Mature	M	Right	Fever, eyelid swelling, exophthalmos, limited eye movement	Sinusitis	Blood and pus	Vancomycin (6 weeks)	No	External drainage	No	No
9	Gogri et al. (10)	2015	12 days	Mature	F	Right	Fever, eyelid swelling, exophthalmos, limited eye movement	Vertical trans-mission	Pus	Linezolid (unknown)	No	External drainage	No	No
10	Chai-Lee et al. (11)	2017	39 days	Mature	M	Right	Fever, eyelid swelling	Gingivitis	Blood and pus	Cloxacillin, metronidazole, and ceftazidime (unknown), vancomycin (14 days)	No	Endoscopic drainage	No	No
11	Tongbram et al. (12)	2021	1.5 months	/	M	Right	Eyelid swelling, exophthalmos	Upper respiratory tract infection	Pus	Vancomycin and linezolid (unknown)	No	External drainage	No	No
12	Tongbram et al. (12)	2021	1.5 months	/	F	Right	Eyelid swelling, exophthalmos	/	Pus	Vancomycin and linezolid (unknown)	No	External drainage	No	No
13	Tongbram et al. (12)	2021	20 days	/	F	Right	Eyelid swelling, exophthalmos	/	Pus	Vancomycin and linezolid (unknown)	No	External drainage	No	No
14	Present	2023	66 days	Mature	M	Right	Fever, exophthalmos, purulent secretion, limited eye movement	Dacryo-cystitis	Blood and pus	Vancomycin (14 days), linezolid (38 days), meropenem (25 days), cefazoxime (20 days)	No	External drainage	No	No

MRSA, methicillin-resistant *Staphylococcus aureus*; GA, gestational age; d, days; w, weeks; m, months; M, male; F, female.

^a^ Dexamethasone.

## 4. Discussion

### 4.1. Classification and source of infection

Clinical manifestations along with ocular imaging findings are used to diagnose orbital abscess. According to the Chandler classification (13), ocular infections can be categorized as follows: (1) inflammatory edema, (2) orbital cellulitis, (3) subperiosteal abscess, (4) orbital abscess, and (5) cavernous sinus thrombosis. It is common for multiple categories to be present in the same patient. When subperiosteal abscess spreads to the orbit, it can lead to the formation of an orbital abscess. Compared to subperiosteal abscess, orbital abscess is associated with higher orbital pressure, resulting in more severe symptoms such as swelling of the eyeball and limited eye movement. Worse still, the optic nerve can be negatively impacted. Unless promptly curbed, the infection may continue progressing and ultimately invade the brain, which can lead to blindness, cavernous sinus thrombosis, meningitis, brain abscess, and even death. Early detection and effective treatment are crucial for managing orbital abscess, which is a pernicious disease.

Sinusitis is the main source of ocular infection, other sources of infection include eyelids, oral cavity, and middle ear (14). This is consistent with the common sources of infection observed in the infants with MRSA-induced orbital abscess in our literature review. In infants, bacteria can easily enter the orbit through the less-developed ethmoid sinuses, leading to orbital abscess (15). However, in the case of our patient, imaging did not indicate sinusitis. Considering that he had a history of dacryocystitis, the likelihood of dacryocystitis being the source of infection was assumed to be high.

### 4.2. Medical treatment

Intravenous vancomycin remains the first-line treatment for complication-free bacteremia caused by MRSA (16). According to the MRSA treatment guidelines published in 2011 (17), the intravenous injection of vancomycin at a dosage of 15 mg/kg q6h is recommended for severely invasive MRSA infections. For severe infections such as bacteremia, infective endocarditis, osteomyelitis, meningitis, and pneumonia, the recommended blood concentration of vancomycin is 15–20 mg/L. In children with positive blood culture results, the duration of antibiotic treatment is recommended to be 2–6 weeks, which can be adjusted according to the etiology. In children, the kidney clearance rate gradually increases with age and approaches adult levels during puberty. Therefore, in 2020, Rybak et al. proposed the application of the area under the curve (AUC) to calculate antibacterial doses in children; they suggest maintaining an AUC/minimum inhibitory concentration (MIC) ratio of 400–600 for vancomycin to reduce the risk of acute kidney damage while ensuring optimal antibacterial effects (18). However, the emergence of vancomycin-resistant MRSA and the poor tissue permeability of vancomycin have made this antibiotic a second-line drug for the antibacterial treatment of the soft tissues and organs; linezolid, which has a lower molecular weight and a higher tissue concentration than vancomycin, is now considered the first-line drug (19). Linezolid has thus been increasingly used, but it is still controversial whether linezolid should be a go-to antibiotic for MRSA infections, given that when used as the primary antibiotic, linezolid may lead to the emergence of linezolid-resistant MRSA and limit treatment options for glycopeptide-resistant *Staphylococcus (*20). In a meta-analysis comparing vancomycin and linezolid for the treatment of MRSA-related pneumonia, linezolid demonstrated higher clinical cure rates and microbial eradication rates, although the mortality rate did not significantly differ between the 2 drugs (21). Yue et al. (22) also reported higher clinical cure rates and microbial clearance rates with linezolid than with vancomycin during the treatment of MRSA-associated skin and soft-tissue infections. Additionally, in contrast to the vancomycin group, the linezolid group experienced a lower incidence of adverse effects, including vancomycin flushing syndrome, itching, and rash, and had a shorter hospital stay and lower hospital costs (22).

Besides our patient, only 1 patient with infantile orbital abscess was reported to develop complications involving the nervous system and respiratory system (7). That patient initially presented with convulsion, and was discharged with neurological sequelae after anti-infective therapy and external drainage. Our patient was diagnosed with MRSA-induced orbital abscess, accompanied by sepsis, pneumonia, and suppurative meningitis. Hematogenous spread was considered to be the cause of the multiple organ infections in this infant. Prior to receiving the etiological results, we proactively administered vancomycin to control the infection. Since AUC monitoring was not available in our hospital, we used the blood concentration of vancomycin as an indicator of its safety and effectiveness, and maintained a trough vancomycin concentration of 15–20 mg/L. In our patient, the orbital infection and sepsis were effectively controlled soon after the initiation of antibiotic therapy and the performance of surgical drainage; however, the pneumonia and meningitis persisted. The infant's lung infection was aggravated, as indicated by the presence of several areas of consolidation and cavities as well as other typical manifestations of *Staphylococcus aureus* pneumonia. After the blood culture turned negative, we replaced vancomycin with linezolid, which noticeably alleviated the pneumonia and meningitis. From this, we drew the following inferences: in patients with MRSA-induced sepsis or bacteremia accompanied by multi-organ MRSA infection, early intravenous vancomycin administration can help contain the hematogenous spread. Once the patient recovers from the bacteremia or sepsis, switching to linezolid, which has better tissue penetration, can be effective. The joint use of vancomycin and linezolid may have a more potent antimicrobial effect against MRSA infection than either drug alone.

### 4.3. Surgical procedures

Not all patients with orbital abscess require surgery. According to Wong et al. (23), severe protrusion of the eyeball, limited eye movement, impaired vision, intracranial infection, and poor antibiotic effect within the first 48 h are common indications for the surgical drainage of pediatric intraocular abscess. Current surgical drainage methods include endoscopic drainage and external drainage. Occasionally, maxillary sinus ostomy and ethmoidectomy have been used to drain orbital abscesses (24). For infants with minor ocular symptoms and good antibiotic treatment outcomes, simple medical treatment is an option. Of the 14 infants with orbital abscesses triggered by MRSA in our review, only 1 was not treated surgically. This decision was justified by the patient's overall stable condition, the location of the abscess on the inner side of the orbit (near the nose), and the negative blood culture results. Additionally, this patient responded quickly to the antibiotic treatment (7). In contrast, our patient exhibited prominent eye symptoms and a poor mental state, along with sepsis and multiple organ infections, which were clear indications for surgical drainage. After the external drainage surgery, the orbital abscess of this infant was significantly mitigated. Currently, external drainage is commonly used for orbital abscesses; however, endoscopic drainage is expected to become more common in the future due to its minimal invasiveness and limited damage to the peripheral nerves and tissues.

### 4.4. Corticosteroids

Some researchers have suggested that corticosteroids can inhibit the toxic effects of cytokines and other mediators, alleviate tissue edema, and facilitate pus drainage, thereby reducing ocular symptoms (25, 26). However, corticosteroids can also suppress the immune system and increase the risk of infection spread. Therefore, it remains controversial whether corticosteroid therapy should be administered to infants with orbital abscess. Davies et al. (27) recommended starting oral corticosteroids when the CRP ≤ 4 mg/dl. They advised a course of oral prednisone at a dosage of 1 mg/kg for 7 days in a row. This treatment plan was believed to shorten the hospital stay of patients while reducing the risk of worsening the infection. Pushker et al. (28) have also reported that patients with ocular cellulitis treated with corticosteroids experienced more significant relief from symptoms, including fever, pain, ocular swelling, and limited eye movement, and their vision recovered much earlier compared to those who were treated without corticosteroids; in addition, patients treated with corticosteroids had a much lower duration of intravenous antibiotic treatment and hospital stay. However, other studies have not found a clear correlation between intravenous corticosteroids and treatment outcomes in children with ocular cellulitis (2). Few studies have investigated the efficacy of corticosteroids in treating children with intraocular abscess. In our literature review on infantile orbital abscess caused by MRSA, only 1 patient received corticosteroid therapy, and was successfully cured. The other 13 infants did not receive corticosteroids and also had a good prognosis. We believe that a definite conclusion about the effectiveness of corticosteroids in infantile orbital abscess cannot yet be drawn due to the small number of cases.

## 5. Conclusion

For the management of infantile orbital abscess caused by MRSA, a comprehensive approach involving aggressive anti-infective treatment and timely surgical drainage is crucial. It is necessary to screen for concomitant infections in other organs and promptly control the blood-borne spread of MRSA as early as possible. This multifaceted strategy aims to minimize the incidence of sequelae and complications, ultimately enhancing the prognostic outcomes of patients.

## Data Availability

The original contributions presented in the study are included in the article/Supplementary Material, further inquiries can be directed to the corresponding author.
